# Informal task‐sharing practices in inpatient newborn settings in a low‐income setting—A task analysis approach

**DOI:** 10.1002/nop2.463

**Published:** 2020-02-27

**Authors:** Gregory B. Omondi, Georgina A. V. Murphy, Debra Jackson, Sharon Brownie, Mike English, David Gathara

**Affiliations:** ^1^ KEMRI‐Wellcome Trust Research Programme Nairobi Kenya; ^2^ Nuffield Department of Medicine University of Oxford Oxford UK; ^3^ Faculty of Health University of Technology Sydney NSW Australia; ^4^ School of Medicine Griffith University Nathan Qld Australia; ^5^ PRAXIS Forum Green Templeton College University of Oxford Oxford UK; ^6^ Aga Khan University Hospital Nairobi Kenya

**Keywords:** healthcare assistants, neonatal tasks, task analysis, task sharing, task shifting

## Abstract

**Aim:**

To describe the complexity and criticality of neonatal nursing tasks and existing task‐sharing practices to identify tasks that might be safely shared in inpatient neonatal settings.

**Design:**

We conducted a cross‐sectional study in a large geographically dispersed sample using the STROBE guidelines.

**Methods:**

We used a task analysis approach to describe the complexity/criticality of neonatal nursing tasks and to explore the nature of task sharing using data from structured, self‐administered questionnaires. Data was collected between 26th April and 22nd August 2017.

**Results:**

Thirty‐two facilities were surveyed between 26th April and 22nd August, 2017. Nearly half (42%, 6/14) of the “moderately critical” and “not critical” (41%, 5/11) tasks were ranked as consuming most of the nurses' time and reported as shared with mothers respectively. Most tasks were reported as shared in the public sector than in the private‐not‐for‐profit facilities. This may largely be a response to inadequate nurse staffing, as such, there may be space for considering the future role of health care assistants.

## 1 INTRODUCTION

Neonatal mortality currently accounts for 22 deaths out of 1,000 live births in Kenya, making up nearly half of all child mortality in the country (Murphy, Gathara, et al., 2018). Thus, reducing neonatal mortality is a key policy concern in Kenya in line with meeting the third Sustainable Development Goal (SDG) (Gathara et al., 2018). To achieve this, high‐quality care for sick and hospitalized babies is imperative. For highly dependent populations, the high quality of care needed is labour intensive and requires appropriate and adequate skilled nurse staffing (Murphy, Gathara, et al., 2018). Unfortunately, there are major nursing workforce deficits in Kenyan facilities and while addressing these can be expensive and challenging (Aiken et al., 2017; Wakaba et al., 2014), changes in how care is organized and delivered could reduce avoidable neonatal deaths (Aiken et al., 2014; Butler et al., 2008; Kane, Shamliyan, Mueller, Duval, & Wilt, 2007). Appropriate forms of task sharing offer a possible solution for service improvements in Kenyan hospitals. This can help address workforce shortages, as long as quality of care delivered is maintained. By critically evaluating the extent of informal task sharing in inpatient neonatal settings in Kenya, we show that there is need for defining clear expectations of neonatal nursing care and support the introduction and formalization of a new or different health worker cadre to aid the nurses in delivering the much‐needed care for sick newborns.

## 2 BACKGROUND

In low‐and‐middle‐income countries (LMICs), task shifting or sharing have already been used to increase access to and uptake of health services, particularly in providing community‐based care or use of non‐physician clinicians in the region (Callaghan, Ford, & Schneider, 2010; Campbell & Scott, 2011; Fulton et al., 2011). In Kenya however, there are no legal or policy provisions for task sharing between nurses and less‐skilled workers (lower cadres) to support the provision of inpatient care.

Introducing task sharing inside hospitals to support nurses is a contested idea, especially in clinical areas where issues around skill and quality of care are particularly sensitive, such as in newborn units (Oluoch et al., 2018). For task sharing to be successfully implemented in such settings, it is vital that the approach is aimed at supporting nurses and that the right tasks are shifted/shared in the right manner to eliminate any potential compromise on quality. The nature of tasks to be shared could be informed by an understanding of those activities that are already subject to informal task shifting/sharing and/or those regarded as time‐consuming but requiring less of a specialized skill set (dos Santos et al., 2016; Dul et al., 2012).

We, therefore, sought to describe the complexity and criticality of neonatal nursing tasks and existing task‐sharing practices to identify tasks that might be safely shared in inpatient neonatal settings to support the work of professional nurses. We theorized that our findings could also potentially inform how nursing care provision could be optimized to achieve and maintain the high standards of quality of care that are required through an informed reorganization of tasks and duties (Murphy, Gathara, et al., 2018). We aimed to achieve this by using task analysis methods (Annett & Duncan, 1967; Hart, Carr, & Fullerton, 2016; Moore 1985).

## 3 THE STUDY

### 3.1 Design

As our main aim was to canvas a wide variety of opinions, subsequently, we conducted a cross‐sectional study in a large geographically dispersed sample using two different survey questionnaires, the facility survey tool and the nurse survey tool (See File S2 and S3) following the STROBE guidelines (See File S1).

### 3.2 Methods

#### 3.2.1 Selection of facilities

**Figure 1:**
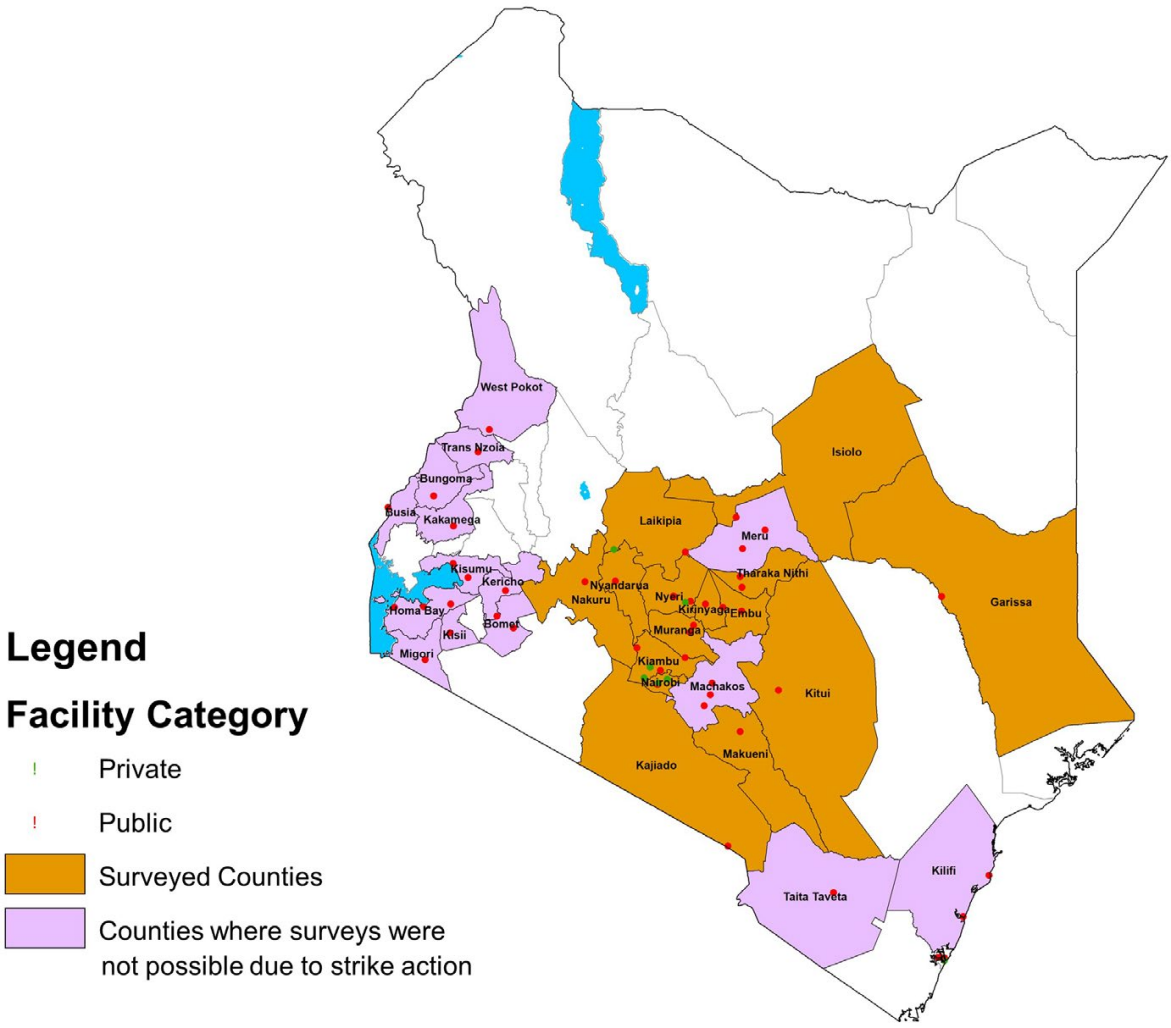
Map of Kenya marking location of the health facilities in the proposed counties for the study and the counties surveyed

In Kenya, as in many LMIC, poor postal services, limited use of personal email and difficulties accessing a comprehensive register of nurses' individual contact details precluded many forms of survey. Therefore, we worked in partnership with the National Nurses Association of Kenya (NNAK) that has 55 branch offices or affiliated representatives in different health facilities in Kenya. These branches cover 31 of Kenya's 47 counties (Figure 1) and are linked to 7 private and 48 public facilities. To these, we added 5 more health facilities (3 public and 2 private‐not‐for‐profit) from Nairobi City County that had taken part in earlier studies evaluating newborn care services (Murphy, Gathara, et al., 2018). All 60 facilities identified in this way provided 24/7 inpatient neonatal care in a functional NBU and were approached to participate in the survey. Facilities were grouped into four geographical regions with a plan for rolling out the survey across these regions sequentially: Nairobi, Central, Western and Coast. However, a 6‐month‐long strike (June to November 2017) prevented us from progressing to include Western and Coast regions (Irimu et al., 2018).

#### 3.2.2 Survey tools

To identify relevant neonatal nursing tasks, we explored literature of prior task analyses and listed potential task items. Selection of candidate task items for possible use in the Kenyan context was then informed by recent, draft recommendations on minimum standards of neonatal nursing in Kenya created by local experts using a consensus process (see File S4; Murphy, Omondi, et al., 2018). Together with a panel of 12 senior nurses during a one‐day workshop, 38 tasks were selected and grouped into 10 domains, with each domain containing between 1–8 tasks (Table 1).

**Table 1:** List of tasks used in the facility and nursing level survey for sharing and difficulty/criticality level responses

Domain	Tasks	Domain	Tasks	Domain	Tasks
Patient assessment and monitoring	Assessing patients during admission and preparing a care plan	Interventions/Investigations	Collecting urine/stool samples	Medication	Oral drug preparation
	6 hourly assessment of clinical status of baby		Giving Vitamin K		IV drug preparation
	Monitoring vital signs of baby 4–6 hourly		Routine cord care		Oral drug administration
	Weighing of baby daily/ on alternate days		Immunization		IV drug administration and cannula care
	Incubator monitoring and settings		Dressing changes		Pre‐discharge counselling on care
			Escorting patients to lab/theatre/X‐ray		Ordering for drugs and non‐pharmaceuticals
			Phototherapy support such as checking exposure and fixing eye pads		
Feeding	Milk preparation and storage	Counselling/Support	Support for Kangaroo mother care	Documentation	Documenting general nursing care in cardex
	NG tube insertion		Counselling on family planning		Handover of patients
	NG tube feeding (3 hourly)		Support with expressing breast milk		Discharge and admission registration
	Cup and spoon feeding (3 hourly)		Counselling on HIV/ STI prevention		
	Checking residual gastric volumes				
	Feeding chart documentation				
Oxygen	Fixing oxygen prongs/nasal catheter	Infection control	Hand washing between patients	Input/Output monitoring	Preparing and administering IV fluids
	Documenting oxygen treatment		Incubator care/ cot cleaning		Documenting input of IV fluids and urine output
Communication	Providing input to medical ward rounds				

Each facility received the facility survey tool, which was completed by the facility's representative, most of whom were senior nurses, providing details on standard practices in the facility relating to sharing of tasks. Each representative was then given between 10–20 of the nurse survey tool to capture the nurses' personal perspectives on the criticality and difficulty of tasks and how much time certain tasks took to implement.

The facility survey tool focused on gathering opinions of nurses' work from a facility's perspective. The focus of enquiry was on the 38 tasks and how they were commonly shared with either the mothers, patient attendants or casuals, in the respective facility's newborn units. We defined mothers as the guardian looking after the baby in the health facility, patient attendants as non‐professional persons who have undergone very limited healthcare training and who are employed in the facility to assist nurses in delivering care to patients and casuals as non‐professional personnel contracted to provide auxiliary services such as cleaning in the facility rather than patient care.

The second tool, the nurse survey tool, was designed for multiple individual nurse respondents from a facility. It focused on the same 38 tasks but participants were asked to rate them on two dimensions; level of criticality and level of difficulty, both on three‐point Likert scales (not difficult/critical, moderately difficult/critical and very difficult/critical) used in previous task analysis surveys (Hart et al., 2016). In addition, a subset of 16 tasks were identified as they were considered likely to be the most time‐consuming. Respondents were asked to estimate, on a Likert scale, the proportion of time they spent on each of these 16 tasks on a typical shift. The Likert scale had five response choices labelled as the percentage of time they might consume on a typical shift (<10%, 11%–25%, 26%–50%, 51%–75%, >75%).

Both the facility and individual nurse survey tool were piloted with nurses from a national referral hospital in a department that did not form part of the study population. Revisions were then made to the initial study tools to establish format and content validity.

#### 3.2.3 Respondents

For the facility level survey tool, we asked that one questionnaire be filled by the NNAK representative (a nurse working in the facility) or a specific nominated facility representative where the NNAK contact person had no recent experience of care on the neonatal unit in the hospital. These representatives completed their facility questionnaires at the end of a one‐day training (see below). The target population for our nurse survey was nurses who worked in study facilities and provided inpatient care for sick newborns in the neonatal, paediatric or maternity units/wards or those who had done so within 2 years at the time of questionnaire distribution (it is not uncommon in Kenya for nurses to be rotated to new units every 1–2 years). Thus, nurses whose experience was only of routine postnatal care to well babies or outpatient care were excluded. We included in the sample nurses who were ward in‐charges and staff nurses, as well as locum nurses and interns, who had worked for at least 2 weeks in inpatient neonatal care settings for sick newborns prior to the data collection exercise. Facility representatives were asked to identify at least 10 eligible nurses and invite them to complete a questionnaire voluntarily. After completion and return of the questionnaires, all nurses were given a Kenyan Paediatric Protocol Booklet as a token of appreciation.

#### 3.2.4 Training

The study team prepared a written manual covering all aspects of the study including standard operating procedures for distributing, completing and checking questionnaires. The nominated facility representative/coordinator was invited for a one‐day training workshop for training on the study processes. For those representatives that were unable to attend the 1‐day training, the same training was provided by a researcher at their facilities. The voluntary nature of participating in the study was emphasized during training. All these wore done to address any potential bias that might occur during the data collection process.

#### 3.2.5 Sample size and data management

Our intention was to include 60 facilities resulting in 60 facility questionnaires and seek individual survey responses from 10 nurses per facility. From a potential sample size of 600 questionnaires, we assumed a non‐response rate of up to 40% for these individual responses, as reported in literature of similar work, that would result in 360 completed questionnaires, deemed sufficient for simple descriptive analysis (Gacula & Rutenbeck 2006).

Completed questionnaires were sent by courier back to the research team. On receipt of the questionnaires, the researchers counterchecked completeness and appropriateness of the information contained. Any incomplete questionnaires (questionnaires with missing data) were excluded from the analysis. Final data were imported into R‐Program version 3 for analysis.

#### 3.2.6 Rigour

To ensure rigour in the study and credibility of our findings, we ensured inclusion of health facilities across the different sectors (public, private and private‐not‐for‐profit). Our selected task items had face validity as these were informed by a panel of stakeholders to ensure relevance to the Kenyan context in addition to pilot testing of tools before data collection. During data collection, quality assurance was undertaken for 5% of the nurse survey respondents who were called to verify whether they participated/responded to the questionnaire. To enhance on data quality, data from the facility and nursing level surveys were double entered into specific Research Data Capture (REDCap) tools by two data clerks and verified by the researchers (GO and DG).

### 3.3 Analysis

Data from the facility level survey and from the nursing level survey were analysed separately. Task‐sharing patterns were examined in aggregate and described across the three sectors (public, private‐not‐for‐profit) and the tasks most commonly shared noted.

Sensitivity analysis showed high correlation coefficient (>.8) when mean scores were compared by sector (public vs. private vs. faith‐based). Thus, data are presented as pooled across the sectors.

We then used the task‐specific mean scores to rank the tasks in descending order in each of the areas of criticality, difficulty and time allocation. Using the ranked lists, we grouped the tasks into tertiles (upper, middle and lower) representing tasks on which nurses reported spending large, moderate or smaller amounts of their time or reported tasks to be most to least critical/difficult. We used these tertiles to summarize nurses' opinions and to explore relationships between criticality, difficulty and time allocation, further cross‐referencing these findings with facility level task sharing findings.

### 3.4 Ethics

Ethical approval for this study was granted by the relevant ethics review committee under the protocol No.3366. Written informed consent was not sought during this study, instead, completion of the questionnaire and voluntarily returning it was interpreted as provision of consent. However, the questionnaire handed over to the participants included a cover information, typical to the information provided in a consent form information sheet, explaining that their participation was completely voluntary and that there would be no negative repercussions from participating, declining to participate or giving a particular response and how their data would be confidentially stored and used. The participants were encouraged to remove and keep this information sheet.

## 4 RESULTS

### 4.1 Facility survey

Survey tools were distributed to 13 of the 31 originally intended counties, and facility responses were obtained from 32/60 eligible facilities (see Figure 1) cutting across public (*N* = 16), private (*N* = 13) and mission/faith‐based sectors (*N* = 3) (with a 100% response rate for those facilities approached).

Table 2 lists the tasks that at least 6/32 representatives reported as most commonly shared with the mothers, patient attendants and casuals in their facilities.

**Table 2:** Showing tasks reported as shared with the mothers, patient attendants and casuals by approximately 6 or more of the representatives^a^

	Proportion of representatives who reported these tasks are shared with mothers, patient attendants and casuals in their facilities (%)
*Shared with mothers*	
Cup and spoon feeding	22/32 (69)
Routine cord care	17/32 (53)
NG tube feeding	13/32 (41)
Oral drug administration	10/32 (31)
Phototherapy support such as checking exposure and fixing eye pads	7/32 (22)
Checking residual gastric volumes	6/32 (19)
Cot cleaning	6/32 (19)
Escorting stable patients to lab/theatre/X‐ray	6/32 (19)
Feeding chart documentation	6/32 (19)
Support for kangaroo mother care	6/32 (19)
Weighing of baby daily/on alternate days	6/32 (19)
*Shared with patient attendants*	
Cot cleaning	14/32 (44)
Escorting patients to lab/theatre/X‐ray	13/32 (41)
Incubator care and cleaning	12/32 (38)
Phototherapy support such as checking exposure and fixing eye pads	7/32 (22)
Cup and spoon feeding	6/32 (19)
*Shared with casuals*	
Cot cleaning	15/32 (47)
Incubator care and cleaning	10/32 (31)
Escorting patients to lab/theatre/X‐ray	9/32 (28)

a These results show what happens routinely in the facilities and the analysis was based on pooled data.

Public sector facilities were leading in the number of tasks reported as shared with the mothers, patient attendants and casuals whereas fewer tasks were reported to be shared in the mission facilities. At least one representative in the public, private and mission sectors reported sharing 27/38 (71.05%), 19/38 (50.00%) and 7/38(18.42%) of the tasks with patient attendants respectively whereas 23/38 (60.53%), 15/38 (39.47%) and 12(31.58%) of the tasks were reported as shared with the mothers by at least one representative from the public, private and mission sector respectively.

#### 4.1.1 Nurses' survey

A total of 632 questionnaires were distributed and 461 nurses returned completed questionnaires, a response rate of 73% (median number of questionnaires returned per facility was 13, IQR 8, 34). The demographics of the respondents are summarized, stratified by sector, in Table 3.

**Table 3:** Demographics of nurses providing 24/7 inpatient neonatal care from the nursing level survey

	Public (*N* = 295)	Private (*N* = 128)	Mission (*N* = 38)	Totals (*N* = 461)	Proportions (%)
*Gender*					
Female	250	11	33	294	63.8
Male	43	15	5	63	13.7
*Age*					
<40	154	102	27	283	61.4
≥40	132	20	11	163	35.3
*Education*					
Kenya Registered Community Health Nurse (KRCHN)	260	123	30	413	89.6
Enrolled nurse	34	4	8	46	10.0
*Primary place(s) of work*					
Newborn Unit	108	35	2	145	31.5
Paediatric Unit	75	23	12	110	23.9
Maternity Unit	51	11	9	71	15.4
Newborn & Maternity Unit	39	44	12	95	20.6
Other places^a^	21	14	3	38	8.3
*Designation*					
In‐charge	32	13	2	47	10.2
Staff nurse	238	95	32	365	79.2
Locum nurse	8	8	3	19	4.1
Intern	13	1	0	14	3.0

a Other places included combinations of newborn, maternity and paediatric units, that is paediatric and maternity or a combination of all three. Some data do not add up to the total number of participants (461) due to missing data.

Overall, most care providers were female (294/461, 63.8%), aged below 40 years (283/461, 61.4%) and were Kenya Registered Community Health Nurses (KRCHN) (413/461, 89.6%). Most nurses responding were providing care to sick newborns in newborn units (108/461, 23.4%), and most were staff nurses (365/461, 79.2%). Nurses' median total experience providing care to sick newborns within the last two years at the time of the survey was 15 months (IQR 1, 48). Only 31 (6.7%) of all the respondents had any additional specialized training in neonatal or paediatric nursing beyond their basic training. Of the 461 nurses from all sectors, only 12 felt that there were sufficient nurses present all of the time in their respective workstations during a typical shift.

#### 4.1.2 Time consumed by specific tasks

Of the 16 tasks about which we enquired, dealing with emergencies, documentation, vital signs monitoring, attending ward rounds and preparing drugs and feeds were in the top tertile of tasks consuming nurses time in newborn care (Table 4). Such task areas capture several activities. For example, dealing with emergencies may involve resuscitation, efforts to stabilize an acutely ill baby or attending to babies with apnoea. Documentation may involve recording the care given such as writing in the cardex, charting vital signs and feeds and documenting treatments. Infection control may involve such tasks as hand washing between patients, disinfection of the cots and incubators or ensuring that medical waste is sorted and disposed of correctly, while administrative duties may involve duty allocations, leave management and attending meetings, among others. Nurses ranked admission and discharge duties, handover of patients, administrative duties, continuous medical education (CME) attendance and feeding equipment preparation in the bottom tertile (Table 4).

**Table 4:** Distribution of the tasks into tertiles (top, middle and bottom third) based on the rank order of mean responses from 461 nurses on how they report spending their time while caring for sick inpatient newborns

Tasks ranked in the upper third/tertile of the list by nurses for time consumed during implementation	Tasks ranked in the middle third/tertile of the list by nurses for time consumed during implementation	Tasks ranked in the lower third/tertile of the list by nurses for time consumed during implementation
Emergencies	Kangaroo mother care counselling	Admission and discharge
Documentation	Input and output monitoring	Handover
Vital signs monitoring	Infection control	Administrative duties
Ward rounds	Teaching and supervision	Continuous medical education attendance
Drug preparation		Feeding equipment preparation
Feeds preparation		

#### 4.1.3 Combining results from facility and nursing level surveys

Table 5 shows how tasks were characterized by nurses in terms of their tertile for level of difficulty and criticality. Of the five tasks considered to be both “very difficult” and “very critical,” only one, “checking residual gastric volume,” was reported to be shared with the mother by >6/32 of the representatives from the facility level survey. Three of the tasks reported as shared with the mothers by the respondents were characterized as very difficult and not critical, these were “collecting urine/stool,” “support with expressing breast milk” and “support for kangaroo mother care.” Two of the five tasks classified as “not difficult” and “not critical” were reported as shared with the mother, and these were “weighing of baby daily/on alternate days” and “escorting stable patients to laboratory/theatre/X‐ray.” Two of the tasks shared with casuals, “incubator care and cleaning” and “escorting stable patients to lab/theatre/X‐ray” were classified under “moderately difficult” and “very critical” and “not difficult” and “not critical,” respectively. From the nursing level survey, six tasks (Table 5) were considered to be time‐consuming in their implementation (in the top tertile), four of these were classified as moderately difficult and moderately critical whereas two were classified as very difficult and moderately critical. Only two of the six tasks, “milk preparation and storage” and “feeding chart documentation” were reported as being shared with the mother by >6/32 of the representatives from the facility level survey. None of the most time‐consuming tasks were shared with any of the identified groups except with the mother.

**Table 5:** Three by three table showing tasks that fall in the three difficulty/criticality categories and sharing patterns as reported by nurses as well as those tasks that nurses ranked as time‐consuming to implement

	Very critical	Moderately critical	Not critical
Very difficult	IV drug administration and cannula care	Counselling on HIV/STI prevention	Collecting urine/stool ■
	NG tube insertion	Providing input to medical ward rounds ▲	Support with expressing breast milk ■
	Documenting input of IV fluids and urine output ▲	Milk preparation and storage ■ ▲	Counselling on family planning
	Checking residual gastric volumes ■		Support for kangaroo mother care ■
	Assessing patients during admission and preparing a care plan		
Moderately difficult	Hand washing between patients	Incubator monitoring and settings	Ordering for drugs and non‐pharmaceuticals
	Incubator care and cleaning ● □	Cup and spoon feeding ■ □	Oral drug administration ■
	NG tube feeding (3 hourly) ■	Monitoring vital signs of baby 4‐hourly ▲	Dressing changes
	IV drug preparation ▲	Feeding chart documentation ■ ▲	
	Fixing oxygen prongs prongs/nasal catheters	Documenting in cardex▲	
		Documenting oxygen treatment ▲	
Not difficult	Phototherapy support such as checking exposure and fixing eye pad ■ □	Routine cord care ■	Weighing of baby daily/on alternate days ■
	Preparing and administering IV fluids ▲	Giving vitamin K	Escorting stable patients to lab/theatre/X‐ray ■ ● □
		Immunization	Oral drug preparation ▲
		6 hourly assessment of clinical status of baby	Pre‐discharge counselling
		Handover of patients	Discharge and admission registration

Note

■: Tasks reported as shared with mothers by >6/32 of the representative from the facility level survey.

●: Tasks reported as shared with casual by >6/32 of the representative from the facility level survey.

□: Tasks reported as shared with patient attendants by >6/32 of the representatives from the facility level survey.

▲: Tasks in the top tertile for “time spent” component of the nursing level survey.

## 5 DISCUSSION

Healthcare workforce shortages are a persistent problem, especially in LMIC that are expected to need 12 million additional workers by 2030 (WHO, 2016). Increasing healthcare workers' numbers and financing their salaries will be major challenges for countries going forward. Task sharing may have a role as one part of a comprehensive solution to these problems and as a means to support well‐trained professionals to focus on delivering highly skilled aspects of health care and reduce the waste of expert nursing time in low skill activities (Campbell & Scott, 2011; Fulton et al., 2011; WHO,^2010^). Task shifting from professionals to community health workers who have short, focused forms of training is already well established in many LMIC as a means to improve coverage with preventive and basic curative care (Fulton et al., 2011). In Kenya as in many other LMIC, there is also a long history of task sharing between different professionals. In some areas, for example, non‐physician clinicians provide most anaesthesia services and nurses provide most curative care in primary care settings including tasks such as prescribing drugs (Dgedge et al., 2014; Pereira et al., 2007; Republic of Kenya, 2017).

Our data confirm previous anecdotal observations that in Kenya task sharing is already happening informally in facilities providing inpatient care to sick and hospitalized babies. This appears to be an informal solution by the nurses working in the public sector who are trying to provide quality care to this highly dependent patient group even in the face of persistent and unfavourable nurse to patient ratios (sometimes as high as 1 nurse to 15 or even more babies) (Aluvaala et al., 2015; Murphy, Gathara, et al., 2018). There is no legal provision for such task sharing in this setting and in fact the use of nurse aides was deliberately banned in the public sector approximately 20 years ago (Oluoch et al., 2018). However, nurses in these settings often report sharing tasks with mothers of babies in the ward. Ethnographic work indicates that tasks may be delegated to a mother to undertake on her own baby and also delegated to “expert mothers” who, after some days on the newborn wards, can coach less experienced mothers to complete tasks (Nzinga, McKnight, Jepkosgei, & English, 2019). The range of tasks reported to be delegated to mothers includes: feeding (NGT feeding or cup and spoon feeding), weighing of babies, routine application of chlorhexidine and other forms of cord care, feeding chart documentation, oral drug administration and collecting urine and stool samples among other tasks. Expert mothers in particular supported tasks such as expressing breast milk and kangaroo mother care (KMC) (Omondi et al., 2018). The involvement of mothers in these ways might be thought of as helping achieve a family‐centred approach to care which may have considerable benefits. However, an alternative view may be that maternal involvement is more of a coping mechanism in the face of the nursing workforce shortages as there appears to be little education, counselling or supervision of mothers' work on these wards (Nzinga et al., 2019).

The shortage of healthcare workers, especially nurses, is not a local or regional problem but a global one. The increasing pressure on both primary and secondary services in high‐income countries due to shortages of skilled healthcare workforce has been widely documented. These challenges have seen the introduction of healthcare assistants/nurse aids in such healthcare settings to augment the nursing workforce through delegation, extension and even introduction of new roles (Bosley & Dale, 2008; Sibbald, Shen, & Mcbride, 2004). In the UK, for example, it is projected that more support staff will be needed to relieve nurses of routine healthcare tasks, whereas in other high‐income settings such as the US, Finland and China, more staff are being adopted to take up support roles both in hospital settings and in home care settings. Such support roles include bathing patients, monitoring and observing them as well as engaging actively in communicating with patients and their relatives, all of which contribute to good quality nursing care (Ingleton, Chatwin, Seymour, & Payne, 2011; Thornley, 2000).

In the context of neonatal wards in Kenya, nurses reported that there are nine tasks considered less difficult that take up a large proportion of the nurses' time. These tasks could potentially be shared with a lower level cadre, allowing nurses to spend more time on other tasks that could not safely be shared with others. Activities that could potentially be shared include: cup and spoon feeding; monitoring vital signs of a baby 4‐hourly; feeding chart documentation; routine cord care; weighing of baby daily/on alternate days; oral drug preparation; escorting stable patients to lab/theatre/X‐ray and phototherapy support such as checking exposure and fixing eye pads.

Despite their potential to improving the quality of inpatient nursing care, healthcare assistants (HCAs) have not been formally recognized in most settings (Hewko et al., 2015). Our findings show that sharing tasks with “patient attendants” and casual workers is informally happening. Formal introduction and implementation of HCAs in LMICs might provide a legal and more regulated way of delegating tasks to non‐nursing staff who can support family‐centred care and take on other auxiliary nursing duties. At the moment in Kenya, there is no legal provision guiding their role in inpatient nursing care and they do not have any recognizable training in public health facilities (Republic of Kenya, 2017). However, implementation of a new cadre must not add to the already high workload of the few, already somewhat beleaguered nurses in such contexts (Omondi et al., 2018). Thus, these scope of practice, training and regulation should be carefully considered and outlined in the appropriate policy provisions.

### 5.1 Limitations

To our knowledge, this is the first study to employ task analysis survey methods in neonatal inpatient health facilities to examine nurses' perceptions of their work in newborn units in Kenya and sub‐Saharan Africa. Survey methods have the advantage of allowing a large number of respondents in a limited time within manageable budgetary allocations. Working with the NNAK made such an approach possible. There are, however, disadvantages to using these methods. Using a Likert scale to collect information may make explicitly distinguishing responses difficult. The responses are also limited to the contexts/themes provided in the survey tool. There is also the inherent possibility of collusion of two or more people in completing questionnaires. For this study, we conducted extensive training with the representatives and ensured that each representative was able to articulate well the purpose of the survey and how to fill in the questionnaire, including encouraging the respondents to avoid collusion and emphasizing individuality and confidentiality. During analysis, the use of tertiles for categorizing tasks is a relatively crude approach and may not give a true picture of the ranking of tasks in the presented realms in this paper. The findings are subjective, and the summary of results hides variation in responses for the nurse survey and behaviours for facility survey. Due to unprecedented disruption of service in the public healthcare sector, we were not able to achieve the sample size we had planned. Also, not only was the desired sample size not obtained but a convenience sample was used which may not be representative of all nurses in the facility, sector or country. Nonetheless, we believe that the health facilities included in this study provide useful information to inform discussions on task sharing.

## 6 CONCLUSION

Nurses report sharing an extensive list of tasks identified by senior nurses as formally part of nursing work. Most sharing is reported to be with mothers, but this appears largely to be a response to inadequate nursing numbers rather than as part of a mentorship approach underpinning family‐based care. Tasks are also shared with casuals and patient attendants but there is no legal or policy provision for this in inpatient settings in the public sector. As part of strengthening the workforce in Kenya, it is imperative to employ more nurses but there appears to be space for considering a future role of health care assistants. More research is needed to support a legal provision of a new cadre to support nurses in delivering newborn care in inpatient settings.

## 7 RELEVANCE TO CLINICAL PRACTICE

By critically evaluating the extent of informal task sharing in inpatient neonatal settings in Kenya, we show that there is need for defining clear expectations of neonatal nursing care and support the introduction and formalization of a new or different health worker cadre to aid the nurses in delivering the much‐needed care for sick newborns. The finding from this paper could be used to ensure that task sharing is implemented safely, effectively and formally through the introduction of a new and regulated lower health worker cadre.

## CONFLICT OF INTEREST

None declared.

## AUTHOR CONTRIBUTIONS

DG, ME and GM substantially contributed to the conception and designed the study. GBO and DG made substantial contributions to the acquisition of data by leading data collection activities with the subject matter experts. GBO, DG and ME contributed substantially to the analysis and interpretation of data. GBO drafted the manuscript with support from DG and ME. All authors (GBO, DG, GM, DJ, SB and ME) substantially contributed to the interpretation of the results and the implications of the findings. DJ, SB, DG and ME were involved in critical review of the manuscript for important intellectual content. All authors critically reviewed drafts of the manuscript and gave final approval before submission.
