# Supplementary material for: Informal task‐sharing practices in inpatient newborn settings in a low‐income setting—A task analysis approach
Source: Nurs Open. 2020 Feb 27;7(3):869–78. doi: 10.1002/nop2.463 (PMC7113512; doi:10.1002/nop2.463)
Supplement: Supplementary file 2 [file NOP2-7-869-s002.pdf]

NEONATAL NURSING TASK ANALYSIS QUESTIONNAIRE

INSTRUCTIONS

1. We would like you to think about your experiences while providing care to sick newborns within the last 6 months in an INPATIENT SETTING and answer the questions provided as honestly as possible while relating to your experiences and what you think is common in your setting while providing care to sick newborns in newborn units/wards.

2. Many of the answers required are around task sharing of routine neonatal tasks and factors that contribute to nursing care being left undone. Please fill in the questionnaire indicating with a tick in the correct column, the answer that best corresponds to your response.

   As an example:
   a) *Please read through the list of neonatal nursing tasks provided and indicate (by ticking in the appropriate column) which tasks you commonly share and with whom you share them.*

Task Area	Apart from the nurse, who else does this task? (tick all that apply)					
	Mother/ family	Trained patient attendant	Casual worker	Student nurse	This task is never shared	This task is not done by nurses
Taking height measurements		✓				
Weighing baby	✓		✓	✓		
Cleaning baby's cord	✓			✓		

3. Please read the questions carefully and ask the study investigators for explanation if you are unsure about anything.

Section A: Task sharing of routine neonatal nursing tasks

Instructions: Please read through the list of neonatal nursing tasks provided and indicate (*by ticking in the appropriate column*) which tasks are commonly shared in your facility/unit and with whom they are shared. If a task is never shared or if a task is not done by nurses, indicate this by ticking in the last two columns appropriately.

Task Area	Apart from the nurse, who else does this task? (*tick all that apply*)					
	Mother/ Family	Trained patient attendant	Casual worker	Student nurse	This task is never shared	This task is not done by nurses
**Patient assessment and monitoring**						
Assessing patients during admission & preparing a care plan						
6 hourly assessment of clinical status of baby						
Monitoring vital signs of baby 4 - 6 hourly						
Weighing of baby daily/ on alternate days						
Incubator monitoring and settings						
**Interventions / Investigations**						
Collecting urine /stool samples						
Giving Vitamin K						
Routine cord care						
Giving OPV/IPV vaccine						
Giving BCG vaccine						
Dressing changes						
Escorting patients to lab/theatre/X-ray						
Phototherapy support (checking exposure, fixing eye pads)						
**Medication**						
Oral drug preparation						
IV drug preparation						
Oral drug administration						
IV drug administration and cannula care						
Pre-discharge counselling on care						
Ordering for drugs and non-pharmaceuticals						

Task Area		Apart from the nurse, who else does this task? (*tick all that apply*)					
		Mother/ family	Trained patient attendant	Casual worker	Student nurse	This task is never shared	This task is not done by nurses
**Oxygen**							
Fixing oxygen prongs/ nasal catheter							
Documenting oxygen treatment							
**Documentation**							
Documenting in cardex							
Handover of patients							
Discharge and admission registration							
**Infection control**							
Incubator care and cleaning							
Cot cleaning							
**Counselling/Support**							
Support for Kangaroo-mother care							
Counselling on family planning							
Counselling on breastfeeding & support for expressing breast milk							
Counselling on HIV / STI prevention							
**Communication**							
Providing input to medical ward rounds							
**Feeding**							
Milk preparation and storage							
NG tube insertion							
NG tube feeding (3 hourly)							
Cup and spoon feeding (3 hourly)							
Checking residual gastric volumes							
Feeding chart documentation							
**Input / Output monitoring**							
Preparing and administering IV fluids							
Documenting input of IV fluids and urine output							

SECTION B: Frequency of nursing tasks provided in the newborn care unit

Nurses often encounter multiple demands on their time and frequently re-prioritize care. In this section we would like to ask you about your own practice. Please think of the infants you cared for within the last 6 months you worked on a newborn unit and the forms of care they received.
For purposes of this questionnaire, please consider how often **you** were able to do the following nursing tasks/activities completely (*as per best practice*) that were necessary given the infants' health status and/ or needs.

We are interested in the nursing tasks that qualified nurses are able to do during their nursing shift. We acknowledge that sometimes nurses delegate tasks to other people, however, for purposes of this questionnaire, if you delegated the task to a student, mother or someone else without a nursing qualification please think of these as tasks you were not able to do.

Please remember that all information you provide is anonymous so answer as honestly as possible.

	How often were you able to do the task as per best practice?	Never	Rarely	Occasionally	Always
1	Baby repositioned at least once every 3 hours				
2	Daily or alternate day weighing of baby				
3	Full incubator cleaning / cot cleaning after baby's discharge				
4	Oral/NGT feed offered on time 3 hourly day and night				
5	Checking residual gastric volumes before feeding				
6	Feeding chart (input) documentation with each feed				
7	Medications administered within 30 minutes of scheduled time				
8	Blood for transfusion verified (i.e. double check with fellow nurse)				
9	Oxygen flow and nasal prongs checked 3 hourly and regulated as prescribed / required				
10	Documenting oxygen treatment in the cardex				
11	Vital signs (temperature, pulse rate and respiratory rate) assessed 6 hourly or as per order and documented on observation chart				
12	Labs/specimens obtained as ordered and within prescribed time				

	How often were <u>you</u> able to do the task as per best practice?	Never	Rarely	Occasionally	Always
13	Baby bathed routinely and/or as needed				
14	Comprehensive physical and physiologic status assessments conducted regularly (at least 6 hourly in very sick babies)				
15	IV cannula site care and assessments at each drug administration				
16	IV fluid intake and urine output monitored and recorded 3-hourly for very sick babies				
17	Regular incubator monitoring and checking of settings				
18	Phototherapy support such as checking exposure to lights and fixing eye pads				
19	All vital information communicated to other staff during handover				
20	Attendance at doctor's ward rounds				
21	Counseling parents so they are included in baby's care – assisted to understand child's condition, actual or planned care and medication				
22	Parents educated about home management of illness, including medications, and general care of infant				
23	Parents prepared for discharge so they are confident in providing care to their baby (including giving ongoing treatment)				
24	Emotional support provided to parents/family				
25	Documentation completed in the cardex as care is provided				
26	Adequately adhere to infection control guidelines (e.g. hand hygiene, aseptic technique, isolation)				

Section C: Reasons for care being left undone/incomplete

Instructions: Thinking about the care that is typically provided in your hospital, please indicate which factors contribute to nursing care being left undone/incomplete

	How important is this reason for preventing all nursing care being provided →	**Very significant** reason	**Moderate** reason	**Minor** reason	**NOT** a reason for undone/incomplete
1	Inadequate number of nurses				
2	Urgent patient situations (e.g. a patient's condition worsening)				
3	Unexpected rise in patient volume and/or severity on the unit				
4	Inadequate number of assistive and/or clerical personnel (e.g. nursing assistants, techs, unit clerks/records people etc.)				
5	Medications were not available when needed				
6	Inadequate hand-over between nurses from previous shift or referring unit				
7	Supplies/ equipment not available when needed				
8	Supplies/ equipment not functioning properly when needed				
9	Lack of back-up support from team members e.g. clinicians, nutritionists				
10	Communication breakdowns with other support departments e.g. radiology, pharmacy, support staff				
11	Communication breakdowns within the nursing team				
12	Communication breakdowns with the medical staff				
13	Nurses lack knowledge and skill in using equipment e.g operating a CPAP machine, using a pulse oximeter				
14	Heavy admission and/or discharge activity				
15	Frequent interruptions				
16	Inadequate documentation what care needs to be completed in the baby's medical record				
17	Parents not present for teaching/counselling				
18	Some care is considered to be of low priority				
19	Inadequate knowledge and experience in newborn care				
20	Inadequate time during shift				

SECTION D: Additional comments

Please provide other comments related to the quality of nursing care provided in your newborn unit or reasons why it may not be optimal here.

SECTION E: Patient load and shift hours

1. **On the last shift you worked providing care to sick newborns, how many newborns were present in the unit where you were providing care?__________**
   - **a.** How many new admissions of newborns were brought to the unit during the shift? ___________________________
   - **b.** How many newborn discharges out of the unit occurred during the shift? ___________________________________
   - **c.** How many nurses were on duty in the unit during the shift? ____

2. **How long does your shift last:**
   - a) during a typical **day** shift [ ] Hours
   - b) during a typical **night** shift [ ] Hours

THANK YOU
