# Supplementary material for: Informal task‐sharing practices in inpatient newborn settings in a low‐income setting—A task analysis approach
Source: Nurs Open. 2020 Feb 27;7(3):869–78. doi: 10.1002/nop2.463 (PMC7113512; doi:10.1002/nop2.463)
Supplement: Supplementary file 3 [file NOP2-7-869-s003.pdf]

NEONATAL NURSING TASK ANALYSIS QUESTIONNAIRE

SECTION A: Neonatal setting

Instructions: *Please fill in the following information about yourself.*

1. **Gender:** Male ☐ Female ☐ Prefer not to say ☐

2. **Age (in years)**

   20 – 29 ☐ 30 – 39 ☐ 40 – 49 ☐ 50 – 59 ☐ ≥60 ☐ Prefer not to say ☐

3. **What is your HIGHEST qualification? *(please tick one box only)***

   Enrolled/certificate nurse ☐
   Diploma nurse ☐
   Higher diploma nurse ☐
   BSN/Degree nurse ☐
   MSN and above ☐

4. **Do you have any specialist training of one year or more in Neonatal Care?**

   YES ☐ **NO** ☐

   If YES please specify____

5. **How much time have you spent providing care to *sick* newborns in an INPATIENT SETTING within the last 2 years? (*please approximate the time in months*)** ☐ **Months**

6. **Please state where you have been providing care to *sick* newborns within the last 6 months in an INPATIENT SETTING and indicate the duration in months that you have been doing so as well as your position in the same setting**

Newborn care setting	Setting (tick only ONE that applies)	Duration (in months)	Position
Newborn unit	☐		In-charge ☐
Maternity unit	☐		Staff nurse ☐
Pediatric unit	☐		Locum nurse ☐
Maternity & newborn unit	☐		Intern ☐
Pediatric & newborn unit	☐		

7. How often do you feel that there are enough nurses in the unit to provide inpatient care to sick newborns?

100% of the time ☐
75% of the time ☐
50% of the time ☐
25% of the time ☐
<10% of the time ☐

Please fill in the rest of the questionnaire based on the experiences you have gained while providing care to sick newborns in an INPATIENT SETTING during typical times within the last 6 months.

SECTION B: Time proportion estimates spent in implementing tasks

Instructions: *Below is a list of general task areas in providing care to sick newborns. We are trying to find out the proportion of time nurses spend in implementing these tasks. First read through the full list of tasks provided then* ***estimate*** *the proportion of* ***time you spend*** *in doing each task during a typical shift within the last six months. Please consider the total time spent doing these tasks during a shift and NOT the time spent per patient.*

For example, if you estimate that routine cord care for the babies in the unit takes about ***30%*** *of your time during a typical shift and cleaning the babies takes about* ***20%****, then indicate these with a tick in the corresponding columns as shown below.*

Task Area	< 10%	11-25%	26-50%	51-75%	>75%	Task done by someone else
Routine cord care			✓			
Cleaning the babies in the unit		✓				

Task Area	< 10%	11-25%	26-50%	51-75%	>75%	Task done by someone else
Preparing and administering medication and IV fluids for the babies in the unit						
Handover of the babies and equipment during shift changes						
Admission and discharge of babies into and out of the neonatal unit						
Vital signs monitoring and regular assessment of condition of the babies in the unit						
Conducting hygiene and infection control activities within the unit e.g. hand washing, proper disposal of waste						
Attending ward rounds and doing follow-up of patient care for the babies e.g. booking tests for babies						
Monitoring input/output of fluids and feeds for the babies in the unit requiring it						
Preparing feeds and feeding the babies via cup or NG tube						
Administrative duties e.g. allocating duties, billing, attending meetings, collecting or ordering supplies etc.						
Counselling mother on KMC and breastfeeding and communicating to mothers on baby's condition/care						
Documenting care and treatment for the babies in the unit						
Dealing with emergencies e.g. resuscitating a baby						
Ensuring the babies are comfortable i.e. bathing babies, preparing their linen and making thier beds						
Cleaning and preparing feeding equipment/utensils for the babies						
Teaching, supervising and mentoring students and other staff						
Attending continuous medical education (CME) meetings						

Please list **ANY OTHER TASKS** that you spend a considerable amount of time doing but have not been listed in SECTION B indicating the proportion of time you spend doing them

Task Area	Proportion of time spent doing the task (%)

SECTION C: Criticality and Level of Difficulty of Neonatal Tasks

Instructions: Below is a table with a list of neonatal nursing tasks performed in the neonatal unit. Indicate, on a scale of **1-3**, **how critical** the performance of the task is to the infant's well-being when performed at the recommended frequency and time. On the same table also indicate, on the same scale indicate **how difficult** the performance of the listed tasks are. Write your rating as a number in the corresponding columns for each task as shown in the example below.

Task Area	How critical is the performance of this task to the infant's wellbeing?	What is the level of difficulty in performing this task?
Fixing intravenous line	2	3
Collecting stool sample from baby	3	1

		CRITCALITY		DIFFICULTY
**Task Area**		**How critical is the performance of this task to the infant's wellbeing?**<br>***1-Not critical***<br>***2-Moderately critical***<br>***3-Very critical***		**How difficult is the performance of this task?**<br>***1-Not difficult***<br>***2-Moderate difficult***<br>***3-Very difficult***
**Patient assessment and monitoring**				
Assessing patients during admission and preparing a care plan				
6 hourly assessment of clinical status of baby				
Monitoring vital signs of baby 4-6 hourly				
Weighing of baby daily/ on alternate days				
Incubator monitoring and settings				
**Interventions / Investigations**				
Collecting urine /stool samples				
Giving Vitamin K				
Routine cord care				
Immunization				
Dressing changes				
Escorting patients to lab/theatre/X-ray				
Phototherapy support such as checking exposure and fixing eye pads				
**Medication**				
Oral drug preparation				
IV drug preparation				
Oral drug administration				
IV drug administration and cannula care				
Pre-discharge counselling on care				
Ordering for drugs and non-pharmaceuticals				

		CRITCALITY		DIFFICULTY
Task Area		How critical is the performance of this task to the infant's wellbeing?<br>1-*Not critical*<br>*2-Moderately critical*<br>*3-Very critical*		How difficult is the performance of this task?<br>1-*Not difficult*<br>*2-Moderate difficult*<br>*3-Very difficult*
**Oxygen**				
Fixing oxygen prongs/nasal catheter				
Documenting oxygen treatment				
**Documentation**				
Documenting in cardex				
Handover of patients				
Discharge and admission registration				
**Infection control**				
Hand washing between patients				
Incubator care / cot cleaning				
**Counselling/Support**				
Support for Kangaroo-mother care				
Counselling on family planning				
Support with expressing breast milk				
Counselling on HIV / STI prevention				
**Communication**				
Providing input to medical ward rounds				
**Feeding**				
Milk preparation and storage				
NG tube insertion				
NG tube feeding (3 hourly)				
Cup and spoon feeding (3 hourly)				
Checking residual gastric volumes				
Feeding chart documentation				
**Input / Output monitoring**				
Preparing and administering IV fluids				
Documenting input of IV fluids and urine output				

SECTION D: Frequency of nursing tasks provided in the newborn care unit

Nurses often encounter multiple demands on their time and frequently re-prioritize care. In this section we would like to ask you about your own practice. Please think of the infants you cared for in the time *within the last 6 months* you worked on a newborn unit and the forms of care they received.
For purposes of this questionnaire, please consider how often were **you** able to do the following nursing tasks/activities completely (*as per best practice*) that were necessary given the infants' health status and/ or needs.

We are interested in the nursing tasks that qualified nurses are able to do during their nursing shift. We acknowledge that sometimes nurses delegate tasks to other people, however, for purposes of this questionnaire, if you delegated the task to a student, mother or someone else without a nursing qualification please think of these as tasks you were not able to do.

Please remember that all information you provide is anonymous so answer as honestly as possible.

	How often were you able to do the task as per best practice?	Never	Rarely	Occasionally	Always
1	Baby repositioned at least once every 3 hours				
2	Daily or alternate day weighing of baby				
3	Full incubator cleaning / cot cleaning after baby's discharge				
4	Oral/NGT feed offered on time 3 hourly day and night				
5	Checking residual gastric volumes before feeding				
6	Feeding chart (input) documentation with each feed				
7	Medications administered within 30 minutes of scheduled time				
8	Blood for transfusion verified (i.e. double check with fellow nurse)				
9	Oxygen flow and nasal prongs checked 3 hourly and regulated as prescribed / required				
10	Documenting oxygen treatment in the cardex				
11	Vital signs (temperature, pulse rate and respiratory rate) assessed 6 hourly or as per order and documented on observation chart				
12	Labs/specimens obtained as ordered and within prescribed time				

	How often were you able to do the task as per best practice?	Never	Rarely	Occasionally	Always
13	Baby bathed routinely and/or as needed				
14	Comprehensive physical and physiologic status assessments conducted regularly (at least 6 hourly in very sick babies)				
15	IV cannula site care and assessments at each drug administration				
16	IV fluid intake and urine output monitored and recorded 3-hourly for very sick babies				
17	Regular incubator monitoring and checking of settings				
18	Phototherapy support such as checking exposure to lights and fixing eye pads				
19	All vital information communicated to other staff during handover				
20	Attendance at doctor's ward rounds				
21	Counseling parents so they are included in baby's care – assisted to understand child's condition, actual or planned care and medication				
22	Parents educated about home management of illness, including medications, and general care of infant				
23	Parents prepared for discharge so they are confident in providing care to their baby (including giving ongoing treatment)				
24	Emotional support provided to parents/family				
25	Documentation completed in the cardex as care is provided				
26	Adequately adhere to infection control guidelines (e.g. hand hygiene, aseptic technique, isolation)				

THANK YOU
