# Supplementary material for: Informal task‐sharing practices in inpatient newborn settings in a low‐income setting—A task analysis approach
Source: Nurs Open. 2020 Feb 27;7(3):869–78. doi: 10.1002/nop2.463 (PMC7113512; doi:10.1002/nop2.463)
Supplement: Supplementary file 4 [file NOP2-7-869-s004.docx]

Neonatal Nursing Tasks

| **Task Area** | **Task done by** | **Frequency (Minimum daily requirement)** |
| --- | --- | --- |
| Admission nursing history, clinical evaluation and vital signs | Nurse | On admission |
| **Drugs** |  |  |
| Drug preparation | Nurse | As per drug schedule (OD, BD, TDS, QID, PRN) |
| Dilutions (compatibility) | Nurse | As per drug schedule (OD, BD, TDS, QID, PRN) |
| Oral drug administration | Nurse | As per drug schedule (OD, BD, TDS, QID, PRN) |
| IV drug Administration | Nurse | As per drug schedule (OD, BD, TDS, QID, PRN) |
| Cannula patency check | Nurse | As per drug schedule (OD, BD, TDS, QID, PRN) |
| Checking cannula sites | Nurse | During shift changes/twice a day |
| **Regular / routine Vital Signs and monitoring** |  |  |
| Temperature | Nurse | 4 times daily/6 hourly |
| Pulse | Nurse | 4 times daily/6 hourly |
| Respiration | Nurse | 4 times daily/6 hourly |
| Checking and documenting oxygen saturation for babies not on oxygen  (for babies on oxygen see section below) | Nurse | Should be conducted ideally 4 times daily/6 hourly for sick babies not on oxygen |
| KMC babies | Nurse | Twice daily monitoring of temperature, pulse and respiration |
| Well babies/stable babies | Nurse | 12 hourly monitoring |
| **Regular / routine monitoring clinical condition** |  |  |
| Skin color | Nurse | 4 times daily/6 hourly |
| Jaundice | Nurse | 4 times daily/6 hourly |
| Respiratory effort | Nurse | 4 times daily/6 hourly |
| Abdominal distension | Nurse | 4 times daily/6 hourly |
| **Intervention monitoring** |  |  |
| Input / Output - General | Nurse | 4 times daily |
| Input - IV fluids | Nurse/clinician | Specific requirements for each baby/frequency depends on prescription/ infusion rate checked every 3 hours |
| Exchange transfusion progress | Nurse/clinical team | Continuous with clinical team during procedure |
| **Oxygen** |  |  |
| Checking tube position and nostril - care, damage | Nurse | 3hourly |
| Initiating and regulating oxygen Flow | Nurse | As required |
| Documenting oxygen treatment | Nurse/clinician | As required |
| Checking and documenting pulse oximetry | Nurse/clinician | 3 hourly/prn |
| Monitoring / regulating pressure | Nurse/clinician | 3 hourly/prn |
| Checking nose / cleaning airway | Nurse | 3 hourly/prn |
| Checking respiration | Nurse | 3 hourly/prn |
| Checking and changing humidifier | Nurse | As required |
| **CPAP Management** |  |  |
| CPAP machine setup | Nurse/clinician | As required |
| Applying nasal prongs / fixing tubing | Nurse/clinician | As required |
| **Phototherapy** |  |  |
| Checking eyes for damage | Baby's family under supervision by nurse | 4 times daily |
| Skin color | Nurse/mother | 4 times daily |
| Checking exposure / baby positioning | Shared by clinical team | Continuous/6hourly/per shift |
| Fixing eye pad | Baby's family under supervision by nurse | Continuous/6hourly |
| Documenting phototherapy | Nurse/clinical team | Shift change/ continuous |
| **Communication** |  |  |
| Parent - counselling, answering questions about clinical / nursing care | Nurse | Continuous/as required |
| Managing record files | Nurse/clinical team | Continuous/as required |
| Providing input to medical ward rounds | Nurse | During ward rounds |
| **Feeding** |  |  |
| Teaching / counseling on Breast feeding (attachment/suck) | Nurse/nutritionist | On admission and prn/daily |
| Checking feed prescribed/ type of feed (check) | Nurse/mother under supervision | 3 hourly or as per feeding schedule |
| Cup feeding | Nurse/mother under supervision | 8 times daily |
| NG tube feeding / checking nostril | Nurse/mother under supervision | 8 times daily |
| Checking residual gastric volumes (ng aspiration) | Nurse/mother under supervision | 8 times daily |
| Charting feed volumes / times | Nurse/mother under supervision | 8 times daily |
| NG tube insertion | Nurse | As required and replace after every 3 days |
| **Administration/documentation of drugs and vaccines** |  |  |
| Giving Vitamin K | Nurse | At birth/as required |
| Routine cord care - antiseptic application | Nurse/Baby's family | Daily |
| Eye care - routine drops application | Nurse/Baby's family | Daily |
| OPV | Nurse | As required for each baby |
| BCG | Nurse | As required for each baby |
| Treatment sheets review | Nurse/clinician | Daily |
| Incident book | Nurse | As required |
| Updating Mother/child health book : vaccines, weight | Nurse | As required |
| Recording in Drug books | Nurse | As required |
| Billing | Multidisciplinary | As required |
| Recording of Stocks – non pharmaceuticals | Nurse | As required |
| **Counselling /support** |  |  |
| Support for KMC | shared with clinician and family members | As required |
| Kangaroo mother care - supervision | Nurse/clinician | As required |
| Health education and progress | Nurse | As required |
| Post discharge care advice, MCH book | Nurse | On discharge/prn |
| Drugs on discharge | Nurse/pharmacist | On discharge |
| Family planning | Nurse | As required |
| HIV / STI prevention | Nurse/counselor/clinician | As required/as required |
| cot cleaning | Support staff | Daily cleaning and thorough cleaning after discharge of a baby before another uses it and as required |
| Cleaning incubator | Support staff | Daily cleaning and thorough cleaning after discharge of a baby before another uses it and as required |
| **Administering Interventions / Doing Investigations** |  |  |
| Taking venous blood | Nurse/clinician | As required |
| Taking heel-prick blood | Nurse/clinician | As required |
| Collecting urine / stool | Nurse/mother | As required |
| Resuscitation with bag valve mask | Multidisciplinary | As required |
| **Counselling/Support** |  |  |
| Expressing Breast Milk | Nurse/nutritionist | As required |
| Bereavement counselling | Nurse/clinician | As required |
| Last office | Nurse | As required |
| **Documentation** |  |  |
| Notifications – Birth | Nurse | As required |
| Notifications – Death | Clinician/HRIO | As required |
| Baby labels | Nurse | As required |
| Discharge and admission registration | Nurse | As required |
| **Waste management and Infection control** |  |  |
| Hand washing | Multidisciplinary | As required |
| Visitors education / practice (on gowns / shoes / hand hygiene) | Nurse | As required |
| **Drugs** |  |  |
| Cross-checking blood for transfusion with co-worker | Nurse | As required |
| Regular baby checks/care |  |  |
| Changing diapers/checking for stool and urine | Nurse/mother/family | As required |
| Cleaning / bathing / clothing | Family | As required |
| Changing bed linens | Nurse/mother/family | As required |
| Weight | Nurse/clinician | Alternate days |
| Incubator monitoring and settings | Nurse | During shift changes/per shift |
| **Milk preparation / storage** |  |  |
| Formula making | Nurse/nutritionist/mother | 3hourly |
| EBM (Storage and labelling) | Nurse/nutritionist/mother | 3hourly |
| Measuring volumes for individual babies | Nutritionist | Continuous |
| Disinfection of cups | Nurse/patient attendant | 3hourly(after every feed) |
| Input and output documentation(amount that has gone in) | Nurse | 3 hourly |
| Exchange transfusion progress | Nurse/clinical team | Continuous with clinical team during procedure |
| **Blood transfusion Exchange transfusion** |  |  |
| Transfusion chart (patient observations / volume of blood) | Nurse^[[1]](#footnote-1)^ | 1/4 hourly |
| Pre administration check of lab results / medical record | Doctor | As required |
| **Miscellaneous** |  |  |
| Accompaniment to lab/ X-ray/ Theatre for procedure or operation | Nurse | As required |
| Accompaniment on outward referral to another facility | Nurse | As required |
| Wound care - checking / renewing dressings | Nurse | As required |
| Last offices | Nurse | As required |
| Preoperative and Postoperative care | Nurse | As required |
| Assistance with Portable chest X-ray | Nurse/radiology team | As required |
| Preoperative and Postoperative care | Nurse | As required |
| Setting alarms (incubator) | Nurse | As required |
| Equipment checks | Nurse/biomedical team | As per schedule/prn |
| Equipment handover | Nurse | Every shift |

1. Tasks listed as formerly being done by nurses can also be done by students under supervision of a qualified staff confirming that the task has been done correctly and as per hospital policy. However students are not to carry out any tasks for category A babies (babies on oxygen, CPAP and IV fluids) [↑](#footnote-ref-1)
